# Patient-physician communication in the emergency department in Taiwan: physicians’ perspectives

**DOI:** 10.1186/s12913-022-07533-1

**Published:** 2022-02-05

**Authors:** Yi-Fen Wang, Ya-Hui Lee, Chen-Wei Lee, Chien-Hung Hsieh, Yi-Kung Lee

**Affiliations:** 1 Office of Industry-Academia Cooperation, National Yunlin University of Science & Technology, Yunlin, Taiwan; 2 Department of Adult & Continuing Education, National Chung Cheng University, Chiayi, Taiwan; 3 Emergency Department, Dalin Tzu Chi Hospital, Buddhist Tzu Chi Medical Foundation, Chiayi, Taiwan; 4 School of Medicine, Tzu Chi University, Hualien, Taiwan

**Keywords:** Patient-physician communication, Emergency department, Roter interaction analysis system

## Abstract

**Background:**

Effective patient-physician communication promotes trust and understanding between physicians and patients and reduces medical disputes. In this study, the Roter Interaction Analysis System was used to explore physician-patient communication behaviors in the emergency departments of Taiwanese hospitals.

**Method:**

Data was collected from the dialogues between 8 emergency physicians and 54 patients through nonparticipant observation, and 675 pieces of data were quantitatively and qualitatively analyzed.

**Results:**

The results showed that: 1. Emergency physicians’ communication behaviors are task-focused. They usually ask closed-ended questions to collect data to identify the symptoms quickly and provide medical treatment. 2. Socioemotion-oriented physician-patient communication behaviors are less common in the emergency department and only serve as an aid for health education and follow-up. Due to time constraints, it is difficult to establish relationships with patients and evoke their positivity.

**Conclusions:**

It is suggested that future education programs on physician-patient communication in the emergency department should focus on strengthening physicians’ ability to communicate with patients in a more open way. They should adopt socioemotional-oriented communication skills, expressing respect and kindness, and allowing patients to briefly describe their symptoms and participate in the treatment process to achieve physician-patient consensus.

## Background

The purpose of physician-patient communication is to create a good physician-patient relationship, promote mutual understanding between physicians and patients, and reduce medical disputes [1, 2]. However, in the emergency department, where speed and efficiency are emphasized, each patient only has an average of 14 s to speak, and only 16% of patients are asked if they have inquiries or understand the information provided by the hospital [3]. This indicates that physician-patient communication is primarily physician-oriented, and few actually attend to the needs of patients. In this regard, important information cannot be conveyed effectively. In addition, there is always a large number of patients in the emergency department, leading to preoccupation and congestion as well as insufficient human resources, which causes medical personnel to have very short and fleeting fractions of time to make judgments [2–4]. Insufficient communication can easily lead to a tense relationship between physicians and patients [5], which is concomitant with negative patient experience, low career satisfaction as a physician, medical negligence, and other problems [4]. Hence, the establishment of effective physician-patient communication is both a pressing need and challenge for emergency departments.

Good physician-patient communication includes instrumental and emotional behavior. The former includes providing information, enquiring medical history, discussing treatment options, explaining an illness, examining the results, etc. [6, 7]. Provision of information is the most common instrumental communication, accounting for 35.3%, followed by medical history inquiry, accounting for 23%, which mostly consists of closed-ended questions [8]. Emotional communication includes self-introduction, calling the patients by their name, giving encouragement and confidence, expressing friendliness, concern, empathy, etc. [6, 9]. Calling the patients by their name is the most common form, accounting for 71.8% [9]. This communication behavior is mainly conveyed through non-verbal expressions such as intonation, eye contact, posture, laughter, facial expression, touch, and distance. Even though oral communication only accounts for 7% [10, 11], it is a key determinant of patient satisfaction. In addition, the use of words is key to effective communication. Good physician-patient communication depends on physicians’ ability to interpret medical language into everyday language, thereby assisting patients to gain a basic understanding of medical language [5, 12]. However, Bourhis et al. [13] further pointed out that the change of words should also consider the understanding and acceptance of patients to shorten the communication gap between physicians and patients.

The Roter Interaction Analysis System (RIAS) is one of the most common evaluation tools and is widely used to explore outpatient medical services in different departments [14, 15]. MaCarthy et al. [16] analyzed the content of physician-patient dialogues and found that emergency physicians occupied a significantly higher amount of dialogue compared to patients, among which most of the dialogue covered patient education and consultation (34%), followed by stimulating patients’ positivity, building relationships with patients, and collecting data. In the dialogue, life and medical themes (86%) accounted for a much higher proportion than psychosocial and social themes (14%). The patients’ dialogue focused on providing information (47%) and building relationships (45%), while asking questions was only 5%. The results showed that dialogue content was mainly about conveying useful information to the other party in the emergency department, and the scores of patient-centered items were low. However, Pun et al. [2] and Levinson et al. [1] pointed out that physicians only require 30 to 60 s to introduce themselves to patients and ask about the chief complaint, and they should listen attentively, show a respectful and friendly attitude and use a peaceful tone, and ask questions in a timely manner to promote mutual understanding, which can greatly improve the quality of physician-patient communication and interaction. On the other hand, the same effect can be achieved by allowing patients to express their condition fully. Langewitz et al. [17] pointed out that the average time of patients’ free talk was less than 1 min and 40 s, and 78% of patients finished expressing themselves within two minutes. As long as physicians and patients keep an open attitude and participate in the communication process together, a consensus can be achieved [18], and medical disputes can be reduced [1, 2]. Therefore, physician-patient communication is worth promoting in medical institutions. However, studies related to physician-patient communication mostly focus on general outpatient clinics and rarely target the emergency department [2, 3, 19, 20]. Therefore, in this study, Roter Interaction Analysis System was used to explore the modes of physician-patient communication in the emergency departments of Taiwanese hospitals, and the results are expected to contribute to effective physician-patient communication in the emergency department.

### Objectives

This study adopted the Roter Interaction Analysis System to explore physician-patient behaviors in the emergency departments of Taiwanese hospitals. The results serve as a basis for devising recommendations for effective emergency physician-patient communication and education. The objectives of the study include:

1. To explore the communication behaviors of emergency physicians.
2. To provide references for physician-patient communication and education in the emergency department based on the research results.

## Methods/design

In this study, nonparticipant observation was first employed to collect and analyze the inquiry data of physicians based on the dialogues between emergency physicians and patients. This research was conducted by the same observer who observed in the emergency department from July to September 2020 without affecting its operation. Without direct involvement in the emergency department, the researcher systematically observed physician-patient communication according to the research purpose and objectively interpreted the observation records to understand the implications of actual situations or behaviors [21, 22]. The physicians’ speech and dialogue were aided by recording the observations on paper and in audio to improve the accuracy and validity of the observation results.

Next, the researchers objectively identified, coded, and classified the contents of physicians’ communication through quantitative and qualitative analysis. They extracted the meaning units of communication behavior, categorized the units into sub-themes and themes. Finally, they performed statistical analyses to understand the content and propensities of physicians’ communication with patients.

### Design

This study adopted purposive sampling to recruit patients from a regional hospital located in a region in Taiwan with the highest proportion of elderly people (20.25% of the region’s population is over 65 years of age). The hospital is equipped with more than 300 beds, serves as a teaching hospital, has a resident physician training system, can cultivate specialists, and has the workforce required for a regional hospital. Researchers have received complete academic training in qualitative research, and they are experienced in interviewing and observation. As a result, upon gaining approval from the hospital’s ethics committee, the researcher observed the modes of communication of emergency physicians at the hospital through nonparticipant observation. The study period was from August to September 2020, during which 7 observation sessions were conducted, totaling 28.5 h. The observation sessions include weekdays and weekends, and they are often in daytime as the patients and the medical staff on duty are more. The participants consisted of were eight physicians (7 males and 1 female) and 54 patients (33 males and 21 females). The dialogue between the participants was recorded, and the content of physicians’ speech was analyzed. The physicians in this study had working experience in the emergency department from 3 to 19 years. The mean length of working experience was 8 years. The extracted content of the physicians’ speech mainly focused on communicating with patients suffering from mild diseases, which levelled 4–5 in Taiwan Triage and Acuity Scale (TTAS) [23], are mentally and physically stable, able to converse clearly with the physicians, and could return home immediately after their outpatient treatment without being hospitalized. The patient sample consisted of 33 males and 21 females with a mean age of 57 and 52 years. The researchers recorded the conversations between the physicians and the patients during the observations and transcribed the audio recordings of the physicians’ speeches into word-for-word transcripts. The contents of the transcripts were coded with designated code names. The first code name represents the physician, and the second and third code names refer to a patient’s age and gender. For example, E-76-F refers to a conversation between Dr. E and a 76-year-old female patient.

The Roter Interaction Analysis System (RAIS) developed by Cavaco and Roter [24] was used as an observation tool in this study. This tool collects behavioral information covering two aspects: task-focused and socioemotional-focused exchanges, each with detailed items. Task-focused exchanges include data gathering and patient education, and counseling skills. Data gathering is divided into four aspects: closed-ended and open-ended biomedical questions and closed-ended and open-ended lifestyles and psychosocial questions. Patient education and counseling skills are biomedical-themed and lifestyle and psychosocial-themed. Socioemotional exchanges include two kinds of behaviors: relationship building and patient activation [24, 25] (see Table 1).

**Table 1:** Sample of an observation data analysis in this study

Communication behavior of RIAS			Summarized meanings of the study sample
Themes	Sub-themes	Meaning units	
Data gathering	Open-ended questions	medical condition and therapeutic regimen	Where else do you feel pain apart from this side? (H-84-F)
		lifestyle and social psychological	What kind of fruit do you usually eat? How much do you eat? (P-65-F)
	Closed-ended questions	medical condition and therapeutic regimen	Are you allergic to any medications or injections? (L-25-M)
		lifestyle and social psychological	Do you rarely drink water? (L-87-M)

### Data analysis

In this study, the researcher transcribed the collected observation data and audio recordings into word-for-word transcription for analysis. The constant comparison method was adopted to classify similar concepts into the same meaning unit based on the communication content and the number of words and sentences of physicians. The meaning units were then summarized into four levels of sub-themes and themes (see Table 1). Then, the researcher calculated the sum of the pieces of data in the themes and sub-themes and converted them into Z-scores to understand the relative position of each item in the overall communication behavior. If the original score is larger than the average, the Z-score is positive; otherwise, it is negative. The name of a person, place, or institution that would directly or indirectly reveal the interviewee’s identity was presented anonymously in accordance with research ethics. Data analysis and verification in this study were performed through the triangulation method to strengthen the credibility and validity of the results [26].

## Results

The researchers collected the contents of communication between eight emergency department physicians, and 54 patients with mild diseases, 27 of whom were older than 65 years old (accounting for 50%of patients). 18 (66.7%) of the elderly patients were accompanied by family members or caregivers to describe their chief complaint. In terms of the content of physician-patient communication in this study, a total of 675 pieces of data containing the physicians’ speech were collected. It was based on the RIAS; the communications mostly focused on data gathering (420 occurrences) followed by patient education and consultation (171 occurrences), facilitation and patient activation (45 occurrences), and relationship building (39 occurrences). The Z-score distribution ranged from − 0.65492 to 2.47469. “Closed-ended questions for data gathering” was the most common communication behavior, while “positive dialogue for relationship building” was the least used (see Table 2).

**Table 2:** Frequency and Z-scores of communication behaviors categorized in the RIAS

Themes	Frequency	Percentage	Sub-themes	Frequency	Z-score
Data gathering	420	62.22%	Open-ended questions	105	0.37495
			Closed-ended questions	315	2.47469
Patient education and counseling	171	25.33%	Biomedical information	149	0.8149
			Psychosocial information	22	−0.45494
Building a relationship	39	5.78%	Social talk	4	−0.63492
			Positive talk	2	−0.65492
			Negative talk	4	−0.63492
			Emotional talk	29	−0.38495
Facilitation and patient activation	45	6.67%	Participatory facilitators	42	−0.25497
			Procedural talk	3	−0.64492
Total	675 (100%)				

## Discussion

The Roter Interaction Analysis System (RIAS) divides modes of communication into task-focused and socioemotional-focused exchanges. The former refers to the contents that patients need to know. The physicians’ speeches mainly include asking questions and providing information, such as data gathering, patient education, and counseling. The latter refers to the support perceived by the patients, mainly in the form of caring, empathizing, and chatting, including relationship building and facilitation and patient activation [24, 25].

**Fig. 1:**
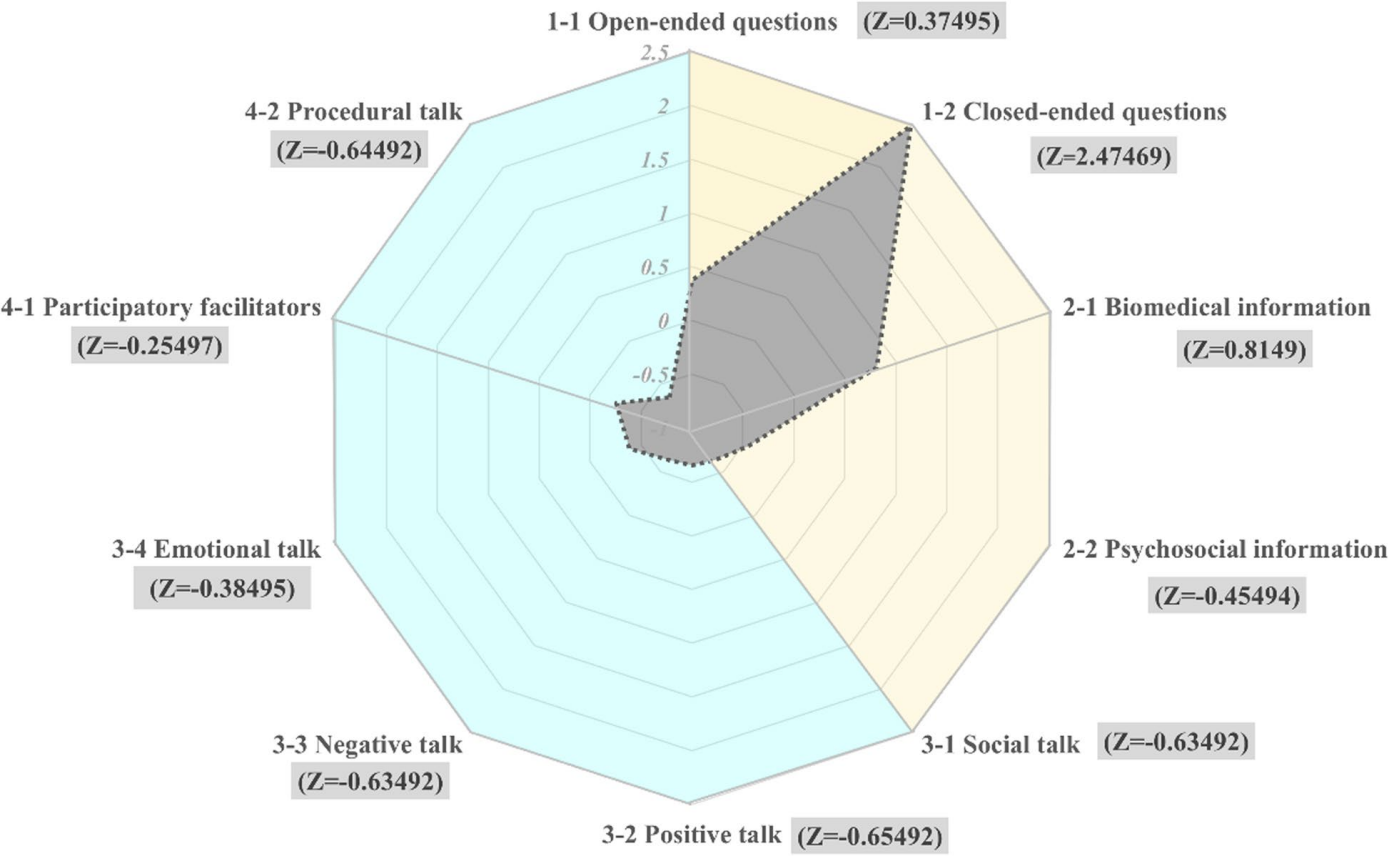
Z-score of RIAS communication behavior distribution

According to the RIAS, this study found that emergency physicians in the Taiwanese regional hospital mainly adopted task-focused communication behaviors (591 occurrences, Z-score = 3.21), asked closed-ended questions for data gathering (315 occurrences, Z-score = 2.47), and focused on biomedical-themed patient education and consulting (149 occurrences, Z-score = 0.81) (Fig. 1). To understand the symptoms of patients in a timely manner, perform medical diagnoses, and administer treatment, the content of physician-patient communication was mainly task-focused, focusing on biomedical themes such as enquiring symptoms, explaining examination results, providing health education and medical advice, explaining the content and method of medication, and performing follow-ups and return visits, etc. [6, 7]. The physicians often asked closed-ended questions beginning with “Have you,” “Will you,” and “Is it.” [2, 4, 8]. Although they used dialects or terms that understandable among elderly patients [5, 12], the patients or their chaperones could only respond with “yes” or “no” answers. The physicians dominated the entire communication process, and patients only waited for questions to be answered briefly [3, 16, 17]. It was found that elderly patients were less likely to ask questions. Their focus is expecting ER doctors to solve the acute symptoms that cause the uncomfortable of their body immediately, shorten the waiting time in the outpatient department, or prevent them from visiting incorrect departments and delaying the diagnosis. Although open-ended questions are better for patients to describe their conditions, emergency physicians often use closed-ended questions to shorten the conversation time with patients in order to obtain straightforward information and offer patients the most appropriate medical services within the shortest time [2, 4].

Medical care emphasizes a patient-centered approach and attaches importance to the feelings and trust of patients, expression of empathy, and provision of emotional support [27]. The process involves understanding the needs of patients, providing information, building relationships, achieving mutual understanding, and joint decision-making [1, 4]. Based on the RIAS model, this study found that there were fewer socioemotional-oriented physician-patient communication behaviors in the emergency department (84 occurrences in total, Z = -3.21), there were only 39 occurrences (Z = -2.31) of relationship-building communication behaviors, and 45 occurrences (Z = -0.90) of facilitation and patient activation communication behaviors (Fig. 1). Due to the need for prompt treatment of patients’ diseases in the emergency department, there is a lack of socioemotional-oriented communication behaviors between emergency physicians and patients, and physicians fail to take into account the patients’ understanding of the communication content, nor attend to the patients’ feelings and needs, etc. [1, 4, 6, 9, 27]. Therefore, it is difficult to include patients in decision-making and for physicians to build trust with patients or their caregivers. Although physician-patient communication also involves psychosocial themes, such as inquiring about the patient’s diet, lifestyle, type of care, etc., they only serve as aids for physicians to evaluate and confirm symptoms and make medical decisions. Therefore, they are often disregarded as items of concern among emergency physicians [16]. Recently, patient-centered approaches have been emphasized in medical care [1, 4, 27]; however, communication skills are not covered in most medical training programs. In addition, emergency departments are associated with negative factors such as high stress and overtime, which reduces the physicians’ initiatives to improve communication skills or build relationships with patients [1, 5]. However, the emergency department’s environment and climate differ from those of the general outpatient department. It is difficult to build relationships and interactions with patients in a short period, which happens to be a feature of physician-patient communication in the emergency department. However, facing the trend of the aging society, the patient-countered conversation method is necessary. Doctors can understand patients’ mental and physical condition through communicating with them and their self-description. And participate in their medical decision to improve the satisfaction of patients.

## Conclusion

This study adopted the RAIS to explore the modes of physician-patient communication in a regional hospital emergency department. The conclusions are as follows: 1. The mode of physician-patient communication in the emergency department is mainly task-focused to identify the disease quickly and provide medical treatment. 2. Closed-ended questions were mostly used for data gathering, which physicians dominated in the dialogues. 3. Patient education and counseling were mainly biomedical-themed, while psychosocial-oriented communication only served as an aid for health education and subsequent follow-ups. 4. Due to time constraints, it is difficult for emergency departments to establish relationships with patients or enhance patients’ positivity. It is suggested that future emergency education programs regarding physician-patient communication in the emergency department should focus on strengthening physicians’ ability to communicate with patients in a more open way and adopt socioemotional-oriented communication skills so that patients are able to describe their conditions within a short period. When physicians exhibit a respectful and friendly demeanor, they would be able to enhance the effectiveness of patient education and counseling and achieve a consensus with patients by including the patients in communication. The research is unable to know if ER doctors’ communicating methods satisfy the idea of patient-countered and the difference from a patient’s perspective, which is the limitation of this study. It is suggested that in light of the increasing number of elderly patients in the future, the results of this study could serve as a reference for developing effective modes of patient-physician communication for elderly people that meet the needs of medical services against the backdrop of an aging society.

## Data Availability

The datasets used and/or analysed during the current study are available from the corresponding author on reasonable request.
